# Cross-cultural translation, adaptation, and validation of the stroke-specific quality of life (SSQOL) scale 2.0 into Amharic language

**DOI:** 10.1186/s12955-023-02092-3

**Published:** 2023-01-23

**Authors:** Dechasa Imiru Wayessa, Mulugeta Bayisa Chala, Solomon Fasika Demissie, Abey Bekele Abebe, Balamurugan Janakiraman, Sisay Deme, Moges Gashaw

**Affiliations:** 1 Department of Physiotherapy, School of Medicine, College of Medicine and Health Sciences, University of Gondar, P.O. Box 196, Gondar, Ethiopia; 2 Institute of Health Policy Management and Evaluation, Dalla Lana School of Public Health, University of Toronto, Ontario, Canada; 3 Department of Research and Faculty of Physiotherapy, Meenakshi Academy of Higher Education and Research (MAHER), Chennai, Tamil Nadu India; 4 Department of Physiotherapy, Faculty of Medical Sciences, Institute of Health, Jimma University, Jimma, Ethiopia

**Keywords:** Translation, Stroke-specific quality of life, Validity, Reliability, Amharic

## Abstract

**Background:**

The stroke-specific quality of life 2.0 (SSQOL 2.0) scale is a valid, reliable instrument which has been widely used as a patients reported outcome measure among stroke survivors. However, the SSQOL scale has not been validated and used in any Ethiopian language. This study aimed to translate, culturally adapt, and test the psychometric properties of the SSQOL scale 2.0 in Amharic, which is the official and working language with about 34 million (23%) speakers in Ethiopia.

**Methods:**

The adapted English version of the SSQOL 2.0 scale was translated into Amharic and then back-translated to English. An expert committee translated and created a final Amharic version of SSQOL (SSQOL-AM) scale. Pre-field testing (pilot and cognitive debriefing) was conducted with 15 post-stroke subjects. The SSQOL-Am was administered to 245 stroke survivors from four referral hospitals to determine the psychometric properties. Cronbach’s alpha and Intra-class correlation coefficient were used to calculate the internal consistency and test–retest reliability, spearman’s correlation for the convergent validity of the SSQOL-Am scale. The Standard Error of Measurement (SEM), Minimum Detectable Change (MDC), Bland Altman Limit of Agreement (LOA), Confirmatory Factor Analysis, and Exploratory Factor Analysis were also determined.

**Results:**

The SSQOL-Am demonstrated excellent test–retest reliability (ICC = 0.93), internal consistency (Cronbach’s alpha = 0.96), SEM 0.857, MDC 1.94, and good LOA. As postulated, the mobility domain of the tool demonstrated a significantly strong correlation with the physical function domain of the SF-36 (rho = 0.70, p < 0.001).

**Conclusions:**

The SSQOL-Am is a valid and reliable outcome measure. The tool can be used in both clinical practice and research purposes with Amharic speaking post-stroke survivors.

**Supplementary Information:**

The online version contains supplementary material available at 10.1186/s12955-023-02092-3.

## Background

Stroke is a major life event and remains the third leading cause of death and disability combined, according to Global Burden of Diseases, 2019 [1]. The detrimental effect on short-term and long-term Health-Related Quality of Life (HRQoL) is known [2, 3]. In 2019, there were 6.5 million deaths from stroke and 143 million lost disability-adjusted life years (DALYs), with a substantial variation between countries. Evidence suggests that globally, the incidence and DALYs related to stroke has increased by 70% and 32%, respectively in the years between 1990 and 2019 [1].

The ultimate goal of stroke rehabilitation is to improve the HRQoL of people with the condition. Hence, it is imperative to explore the impact of the process of planning, evaluating, and rehabilitative intervention on the self-rated HRQoL of stroke survivors [4]. Most outcome measures used in stroke rehabilitation are focused on symptom changes and functional restoration, with less emphasis on patient-centered assessment including subjective well-being and HRQoL [5, 6]. Studies report that there are enormous variations in the self-reported measures addressing different domains of HRQoL. There is variability in the stroke-related quality of life reports due to variations in the outcomes measure, cultural, language, and social differences of the stroke survivors [7, 8].

Several patient-reported outcome measures are used to capture the dimensions of health and quality of life after stroke to supplement the clinical and social decision-making [5, 9]. The stroke specific Quality of Life scale (SSQoL 2.0), developed by Williams et al. 1999, is a widely practiced, well-known, standardized, and comprehensive disease-specific self-reported health related quality of life scale designed to report multiple life impacts of stroke in stroke survivors [10]. The SSQoL was originally derived from the interview of stroke survivors in the USA, the scale has demonstrated good validity and reliability when tested in different types of stroke survivors. The SSQoL contains 49 items and measures 12 domains in life that are impacted by stroke, like self-care, vision, language, mobility, productivity, upper-extremity function, thinking, personality, mood, energy, social, and family roles.

The SSQoL 2.0 has been cross-culturally translated, adapted, and validated for use in several other languages and cultural contexts, with studies in African language under reported [11–21]. To our knowledge, the SSQoL has not been cross-culturally translated, adapted, or used in any of the Ethiopian languages. Amharic language is the official working language with 34 million people speaking this language in Ethiopia [22]. Although stroke is a prevalent health problem in Ethiopia, there is a limited utilization of a multi-dimensional self-reported stroke-specific scale in the country. The availability of a validated Amharic version of the SSQoL 2.0 will surely improve the utility of SSQoL among the clinicians and researchers. Further, the psychometrically tested Amharic version of SSQoL would foster multicenter and multinational collaborative researches on Stroke-related quality of life across the globe. This research aims to translate and culturally adapt SSQoL into Amharic and to evaluate its psychometric properties, including internal consistency, test–retest reliability, convergent validity, and factor structure among stroke survivors.

## Methods

This multi-center-based cross-sectional validation study was conducted in two phases. Phase one was to translate and culturally adapt the SSQoL to the Amharic language (SSQoL-Am). Phase two involved testing the psychometric properties of SSQOL-Am. Ethical clearance was obtained from the Institutional Review Board of the University of Gondar (ref no: SOM1942/03/2020). Permission to conduct this study was sought from all study sites. Written consent was taken from all the participants before their enrollment in the study. This was conducted by following the principles of the Helsinki declaration.

### Phase one translation and cultural adaptation into the Amharic language

The translation and cross-cultural adaptation of SSQoL into the Amharic language was conducted according to Beaton et al.'s five-step guideline for the process of Cross-Cultural Adaption of Self-reported Measures [23].

In Step one, two Amharic native translators (a physiotherapist and a accountant) who were fluent in the English language translated the original SSQoL into Amharic (SSQoL-Am) independently. None of them were informed about the concept and purpose. They produced two forward-translated documents (T1 and T2). The principal investigator mediated the disagreement during consensus of the translations. In Step two, the independently translated SSQoL-T1&T2 documents were shared among the two translators to synthesize and reach on consensus on the Amharic version of the SSQoL-Am (version 1). Any differences in the concepts and/or meaning were sorted through discussions. In step three, the first draft SSQoL-T12 was back-translated to English. This was done by two bilingual independent translators, one of them was a practicing physiotherapist at University of Gondar (UOG) and the other one a PhD student at the Queen’s University, who also have a physiotherapy background in Ethiopia. One of the translators (clinical physiotherapist at University of Gondar) has never used the SSQoL measure, and both the back translator were blinded to the process of forward translation. The back translated English version of SSQOL was checked for corrections in words or phrases, and conceptual equivalence with original version of SSQOL through the discussion, and agreement between the two back translators and the principal investigator (DI). All the three documents were presented to the panel for step four.

In step four, an expert committee was set up to review and discuss all the translated and adapted versions. The panel included all the four translators, a research methodologist and a neuro-physician, both had expertise in outcome research, tool validation and development, a stroke survivor with 3.5 years stroke chronicity, and a native English speaker who is also fluent in the Amharic language. All the panel members were given the original English (version) SSQoL 2.0 scale, T1 & T2, consensus Amharic, and the back translated English versions for review. The panel was assembled to discuss the idiomatic, clarity, relevance, comprehension, response option, review and synthesize the translation and cultural adaptation process in a stepwise manner, and verify the final SSQoL-Am tool for a pre-field testing. Upon the approval of the panel, the questionnaire was piloted with 15 post-stroke survivors at the out-patient unit of the University of Gondar comprehensive specialized hospital (Step five). A cognitive (qualitative) debriefing [24] was conducted with the 15 participants to assess the clarity of the tool, item understandability, language used, cultural appropriateness, and acceptability of the SSQoL-Am. After which the translated and adapted form of the Amharic version of SSQoL-Am was ready for psychometric testing among the Amharic-speaking stroke survivors.

### Phase two: psychometric testing of the SSQoL-Am

#### Study setting and participants

This multicenter study was conducted in the Amhara regional state, two specialized hospitals (University of Gondar comprehensive specialized hospital (UoGCSH) in Gondar and Felege Hiwot referral hospital (FHRH) in Bahir Dar) and two referral hospitals, Dessie referral hospital (DRH) and Debre Berhan referral hospital (DBRH)), these referral centers are the main public health centres of the respective region. The hospitals receives wide spectrum of stroke patients and provide free consultations, medications, medical care, and rehabilitation. All the adult stroke patients who visited the outpatient departments at these hospitals were approached and recruited for the study by convenient sampling. We included Amharic speaking stroke patients with a diagnosis of ischemic or hemorrhagic stroke of not less than one-month duration, both genders, 18 years or older, willing to consent, able to understand and communicate were included. Stroke survivors with dysphasia, intellectual impairments, traumatic head injury, psychiatric diseases, and dementia were excluded. This is in line with the Lin et al. recommendations to improve the generalizability of the findings [25].

Two hundred and forty-five post-stroke survivors were recruited from the physiotherapy units of UoGCSH, FHRH, DRH, and DBRH from February 2021 to May 2021. The required sample was calculated based on the Consensus-based Standard for Selection of Health Measurement Instrument (COSMIN), which proposes at least 5–10 participants per item in the scale [26, 27].

All the participants completed the socio-demographic data, clinical characteristics, SSQoL-Am, and SF-36-Am. Data was collected by the trained physiotherapists working at the study sites in the Amhara region using the interview method. Clinical data, stroke-related characteristics, and physician’s diagnosis were extracted from the patient’s medical chart.

#### Predefined hypotheses

We predefined hypotheses for the direction and magnitude based on the checklist for evaluating the methodological quality of studies on measurement properties of health status instruments [28]. Construct (convergent) validity was considered to be supportive if ≥ 75% of the results were in agreement with our hypotheses. Convergent validity was established by comparing the linear association of each score of SSQoL-Am with the specific domain of SF-36. We expected a moderate positive correlation between the mobility domain of SSQoL-Am and SF-36-Am physical functioning. The correlation coefficient values < 0.5, 0.5–0.69, and ≥ 0.7 were considered weak, moderate, and good correlation, respectively. We also expected weak to moderate positive correlations between the energy domains, work/productivity, family domains of SSQoL-Am, and vitality, emotional domains of SF-36 subscales, similar to the previous studies [18, 19, 29, 30].

#### Patient-reported measures

##### Stroke Specific Quality of Life (SSQoL)

The original SSQoL scale consists of 49 items [10] grouped into 12 domains, each domain containing 3 to 6 items. Each item has a minimum score of ‘1 (meaning the worst outcome) and a maximum score of ‘5’ (meaning the best outcome)’. The purpose of this patient-centered self-reported outcome tool is to measure the health-related quality of life.

##### Short Form Health Survey (SF-36)

The SF-36 with 36 items [31] is intended to measure the quality of life of respondents with certain health conditions [30, 31]. It is also widely used to evaluate the convergent validity of patient-reported outcome measures [14, 16, 18, 30]. The SF-36 is translated, adapted, and validated into Amharic (SF-36-Am) [32] using the Ethiopian population. For the 8 domains of the tool, the items were coded, summed, and transformed into 0 (indicate worst-health related quality of life) to 100 (indicate worst-health related quality of life) scale. The bodily pain subscale was reverse coded into 0–100 to suit the higher value indicating better health [33].

### Reliability

The reliability of the SSQoL-Am was determined by calculating Cronbach’s alpha with an alpha value of > 7.0 assumed acceptable, > 0.8 considered good, and > 0.9 considered excellent for internal consistency [34]. The Amharic version of SSQoL was re-administered on a third of the participants (n = 80) at the interval of 7 days of the first administration to assess its test–retest reliability [35].

### Analysis

Statistical analyses were performed using the Statistical Package for Social Science version 25 (IBM SPSS INC., Chicago IL, USA). Descriptive analysis of socio-demographic and clinical variables was described using mean, standard deviations, interquartile range, and chi-square. Intraclass-correlation coefficient (ICC_agreement_ 2,1) using a two-way random-effects model [36] and Cronbach’s alpha [26] were calculated to assess the test–retest reliability and internal consistency. The Bland–Altman 95% Limit of Agreement (LOA) [37] indicates the magnitude of random changes by systematic variation or random measurement error. The plan was to calculate Pearson correlation coefficients (Rho) between the Amharic version of SSQoL and SF-36 (Am) if the assumptions of normality were met [38] and the Spearman’s correlation coefficients (Rho), when data demonstrated non-normality, the Shapiro–Wilk test of normality, skewness, and kurtosis was conducted to evaluate the normality of the data [39]. A disaggregated analysis of the correlation was performed to explore the difference between gender, acute and chronic stroke, and Hemorrhagic and ischemic stroke categories [40]. Further, the reliability of the tool was assessed by the standard error of measurement (SEM, and minimum detectable change (MDC), by using the formula SEM = SD √ (1-R) and MDC = 1.96 √2 × SEM, respectively.

The floor and ceiling effects of SSQoL-Am were established by calculating the percentage of the respondent’s lowest and highest scores on the tool. The floor and ceiling effects were assumed optimal if it did not exceed 15% [26]. In addition, Confirmatory Factor Analysis (CFA) was done to investigate if SSQoL-Am is a uni-dimensional or two factors model [41], as reported by previous studies [42, 43] with the difference in factor structures using STATA version 14 (College Station, Tx, StataCorp). The model fit of SSQoL-Am was assessed by the goodness of fit index (GFI), adjusted goodness of fit index (AGFI), comparative fit index (CFI), and root mean square error of approximation (RMSEA). The dimensionality of the SSQoL-Am was determined using Kaiser Meyer Olkin (KMO) and Bartlett’s test of sphericity with a retention rule (item value) of Eigenvalue > 1 and scree test plot [44–46]. The EFA of SSQOL-Am was conducted using a Maximum Likelihood with a Varimax rotation model, and extraction was done using a 0.4-factor loading principle [47].

## Results

### Cross-cultural translation and adaptation

The translators and the expert panel reached an agreement during the translation and adaption of the SSQOL in to Amharic and the local context. None of the items in the original scale was removed in either forward or back translations. The socio-demographic and clinical characteristics of the participants for the pilot testing and cognitive debriefing of the pre-final Amharic version are summarized in Table 1. All the participants reported that the tool is easy to understand, convinced, and completed the scale during cognitive debriefing. The expert committee agreed that all the items in the Amharic version translations of the SSQOL scale captured the construct of interest as provided in the English version. It also identified that some words such as ‘‘trouble’’ was inconsistently used in back-translated English versions such as ‘difficulty’, ‘problem/s’, and ‘challenge’. A few some tense discrepancies were also identified between the original English back-translated English version. These discrepancies were resolved and some items were modified by the expert committee.

**Table 1:** Socio-demographic and clinical characteristics of the participants with stroke involved in the pilot testing and cognitive interviews (n = 15)

Characteristics	Frequency n (%)
Age (in years) mean SD	51.33 ± 16.63
Sex	
Male	9 (60)
Female	6 (40)
Residence	
Urban	14 (93.3)
Rural	1 (6.7)
Religion	
Orthodox	12 (80)
Muslim	2 (13.3)
Protestant	1 (6.7)
Marital status	
Single	2 (13.3)
Married	9 (60)
Widowed	2 (13.3)
Divorced	2 (13.3)
Employment status	
Farmer	5 (33.3)
Employed	6 (40)
Retired	4 (26.7)
Stroke types	
Ischemic stroke	8 (53.3)
Hemorrhagic stroke	7 (46.7)
Co-morbidities	
Hypertension	9 (60)
Diabetes mellitus	2 (13.3)
Heart problems	1 (6.7)
Others	3 (20)
Affected side	
Right side	5 (33.3)
Left side	10 (66.7)
Stroke chronicity in month’s Mean (SD)	9.6 (± 10.48)

*SD* standard deviation

Participants involved in the pilot testing and cognitive debriefing interview of the pre-final Amharic version of the scale consisted of 15 (nine male and six female) stroke survivors. The participants mean age was 51.3 ± 16.63 years, with the mean stroke duration 9.60 ± 10.48 months. The scale showed excellent internal consistency during the piloting of the tool with Cronbach’s alpha value of 0.92. During the cognitive debriefing interview, participants were asked about the clarity or ambiguity of the items and response options. They were also asked if all necessary items in respective to their conditions were covered by the scale and whether all the items were relevant to them. The findings show that all the participants understood all items in the pre-final Amharic version, all items were relevant to their condition, and no important part was overlooked. They had clarity and the questionnaire was clear during the cognitive debriefing. However, four male participants recommended that item # 1 in the self-care domain ‘Did you need help preparing food or if you are to prepare food?’ which was translated as “  ” did not apply to them culturally. Their reason was that most Ethiopian men do not traditionally get involved in food preparation or do household activities. We acknowledged the respondent's valuable feedback, but the panel of experts agreed that making such modifications on the scale would make it unsuitable for the new generation and men who help in food preparations. The final version of the SSQoL-Am is available in Additional file 1.

### Psychometric testing

A total of 245 stroke survivors participated in this phase of psychometric testing, with a response rate of 100%. The higher response rate could be because the participants were following physiotherapy treatment in the centres. The mean age of participants was 58.09 ± 13.75 years, and a majority of the participants 189 (77.1%) were married. More than half of the participants, 142 (58%) were males and most (84.5%) of stroke survivors in this study belonged to orthodox Christian. Most of the participants, 74.3% reside in towns and cities. Among all participants, 27.8% were illiterate. The socio-demographic and clinical characteristics of the participants are summarized in Table 2. The mean duration of stroke was 17.5 ± 20.5 months and 147(60%) of participants had experienced right-sided hemiparesis. The majority 166 (67.8%) had an ischemic type of stroke. Most of them (61.2%) had hypertension and about 55% of the participants were diagnosed with stroke since 6 months. The overall mean score of participants of SSQOL-Am was 142.8 ± 30.71 Table 3.

**Table 2:** Socio-demographic and clinical characteristics of stroke survivors for psychometric testing (n = 245)

Characteristics	Acute (n = 109)	Chronic (n = 136)	Total (n = 245)	P
	n (%)	n (%)	n (%)	
Age(mean ± SD)in years	55.1 ± 12.9	60.21 ± 13.0	58.1 ± 13.5	
Sex				
Male	50 (45.9)	92 (67.6)	142 (58)	
Female	59 (54.1)	44 (32.4)	103 (42)	0.01
Residence				
Urban	84 (77.1)	98 (72.1)	182 (74.3)	
Rural	25 (22.9)	38 (27.9)	63 (25.7)	0.37
Educational status				
Uneducated	24 (22.0)	44 (32.4)	68 (27.8)	
Primary school	39 (35.8)	54 (39.7)	93 (37.9)	
Secondary school	24 (22)	22 (16.2)	46 (18.8)	
Tertiary (college & above)	22 (20.2)	16 (11.8)	38 (15.5)	1.36
Religion				
Orthodox Christian	94 (86.2)	113 (83.1)	207 (84.5)	
Muslim	13 (11.9)	21 (15.4)	34 (13.9)	
Protestant	1 (0.9)	2 (1.5)	3 (1.2)	
Catholic	1 (0.9)	0 (0.0)	1 (0.4)	0.57
Marital status				
Single	6 (5.5)	13 (9.6)	19 (7.7)	
Married	93 (85.3)	96 (70.6)	189 (77.1)	
Divorced	4 (3.7)	7 (5.1)	11 (4.5)	
Widowed	6 (5.5)	20 (14.7)	26 (10.6)	0.03
Employment status				
Farmers	5 (4.6)	13 (9.6)	18 (7.3)	
Employed	20 (18.3)	16 (11.8)	36 (14.7)	
Retired	7 (6.4)	22 (16.2)	29 (11.8)	
Business	27 (24.8)	23 (16.9)	50 (20.4)	
Others	50 (45.9)	62 (45.6)	112 (45.7)	0.03
Co-morbidities				
Hypertension	70 (64.3)	85 (62.5)	155 (63.2)	
Diabetes mellitus	13 (11.9)	15 (11)	28 (11.4)	
Heart problems	16 (14.7)	32 (23.5)	48 (19.6)	
Others	10 (9.2)	4 (2.9)	14 (5.7)	0.13
Affected side				
Right	61 (56)	86 (63.2)	147 (60)	
Left	48 (44)	50 (36.8)	98 (40)	0.2
Type of stroke				
Ischemic	72 (66.1)	94 (69.1)	166 (67.8)	
Hemorrhagic	37 (33.9)	42 (30.9)	79 (32.2)	0.6
Stroke duration (month) mean ± SD	2.3 ± 1.56	22.6 ± 12.7	14.65 ± 9.2	0.001

Employed category refers to private/governmental employee

**Table 3:** Reliability (n = 245) of the Amharic version of the SSQoL-Am, internal consistency, floor and ceiling effects, test–retest reliability, and Standard Error of Measurement (SEM), (n = 72), 2021

SSQoL domains (items)	Mean (SD)	Internal consistency Cronbach’s α	Floor and ceiling effects (%)		ICC (95% CI) (n = 72)	SEM
Energy (3)	7.81 (2.67)	0.84	0.8	1.6	0.88 (0.81, 0.92)	0.4
Family (3)	8.48 (2.72)	0.72	1.6	3.3	0.83 (0.73, 0.90)	0.46
Language (5)	16.24 (4.21)	0.93	0.4	1.6	0.88 (0.78, 0.93)	0.44
Mobility (6)	16.78 (4.9)	0.92	0.8	0.4	0.90 (0.85, 0.94)	0.41
Mood (5)	15.36 (4.72)	0.85	1.6	4.5	0.92 (0.84, 0.95)	0.44
Personality (3)	8.85 (3.52)	0.93	4.9	7.3	0.92 (0.85, 0.95)	0.40
Self-care (5)	13.63 (4.3)	0.90	2	1.6	0.91 (0.86, 0.94)	0.40
Social role (5)	12.61 (3.4)	0.78	0.4	0.4	0.90 (0.82, 0.94)	0.37
Thinking (3)	9.87 (2.98)	0.83	1.2	6.1	0.89 (0.81, 0.93)	0.36
UE function (5)	13.69 (4.35)	0.91	1.6	0.8	0.88 (0.81, 0.92)	0.44
Vision (3)	12.87 (2.32)	0.89	0.4	44.1	0.82 (0.72, 0.89)	0.42
Work (3)	6.60 (2.5)	0.92	3.3	2.4	0.89 (0.88, 0.95)	0.38
Total Score (49)	11.9 (3.6)	0.87	1.58	6.18	0.93 (0.88, 0.95)	0.41

*Upper Ext.func* Upper Extremity function, *ICC* Intra-class correlation coefficient

### Reliability of the SSQoL-Am

**Fig. 1:**
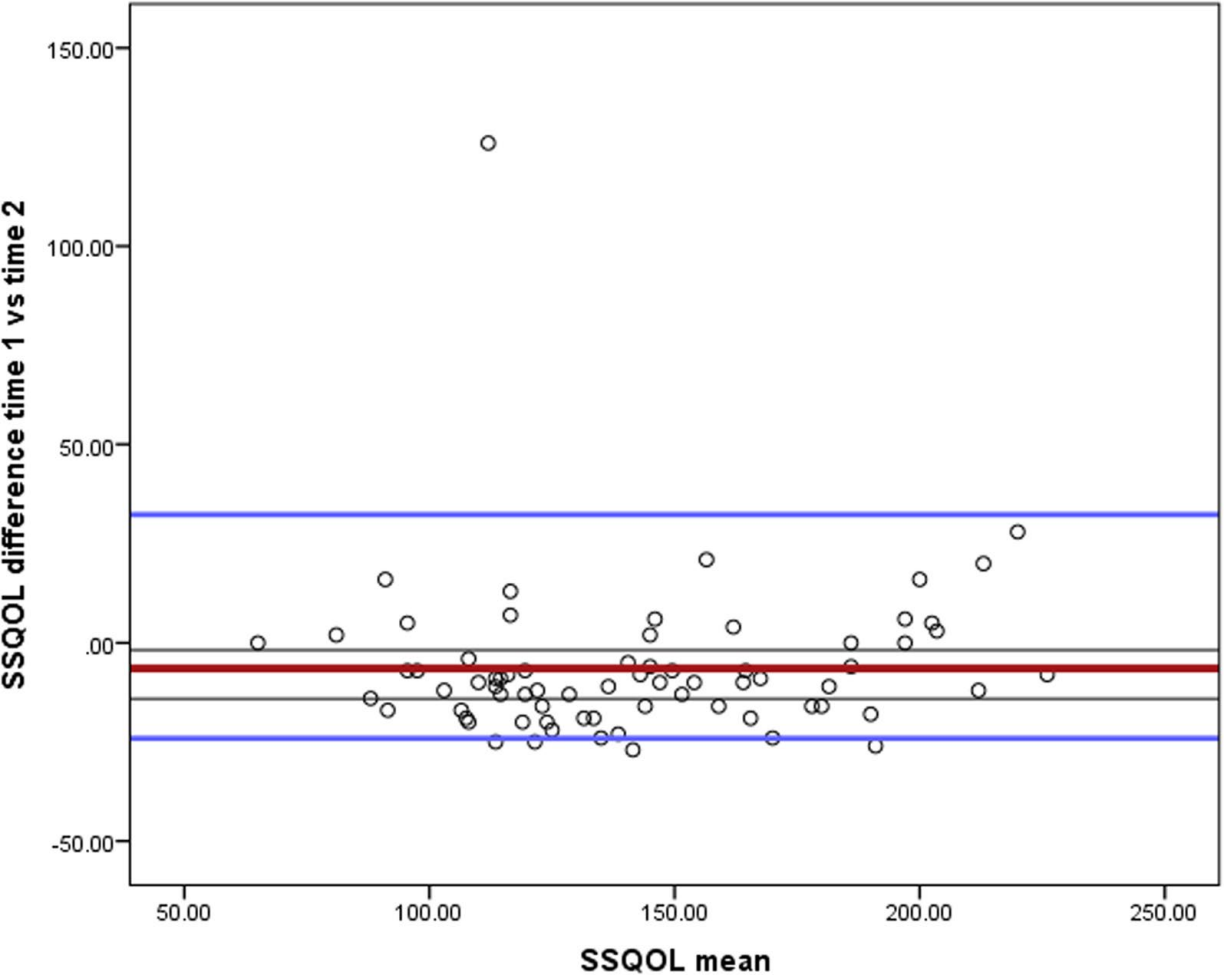
Bland–Altman plot of agreement between test and re-test scores of the SSQOL-Am with bold red line representing the mean of difference, the blue lines representing 95% limits of agreement (LOA) and the grey lines representing the 95% CI of the mean of the difference

The SSQOL-Am scale demonstrated excellent internal consistency with Cronbach’s alpha of 0.96. Table 3 and the 12 domains demonstrated acceptable to excellent internal consistency, with Cronbach’s alpha values ranging from 0.72 to 0.93. The internal consistency, if item deleted ranges from 0.958 to 0.979. Table 4 suggests the internal consistency if the items are deleted. Both language and personality domains had scored the highest among other domains (α = 0.93). SSQoL also demonstrated excellent reliability in the subgroup analysis by gender (male α = 96 vs female α = 96). All domains of the SSQOL-Am, except the vision domain, demonstrated no evidence of ceiling effects. The floor and ceiling effects of the 12- domains of the SSQOL-Am are presented in Table 3. Among the 80 participants who were invited during the re-administration of the SSQOL-Am scale, 72 stroke survivors completed the questionnaire. The test–retest intra-correlation coefficient (ICC) value was 0.93 (95% CI 0.88–0.95) indicating excellent test–retest reliability. The overall standard error of measurement (SEM) and Minimal Detectable Change (MDC) were 0.43 and 1.27, respectively Table 3. In addition, the Bland–Altman plot shows that all the observation except one lie within the limit of agreement (LOA) ± 1.96, ± SD 19.81. Furthermore, and the linear regression model of both measures were non-significant (p = 0.609), indicating no evidence of systematic change between the two measures and no proportional bias in the measures. The 95% CI of LOA between the test–retest scores ranged between − 19.6 and 31.11 (Fig. 1).

**Table 4:** Internal consistency of SSQoL-Am if item deleted

Item-total statistics				
	Scale mean if Item deleted	Scale variance if Item deleted	Corrected item-total Correlation	Cronbach's α if Item deleted
Q1 Energy	142.82	1390.544	.693	.979
Q2 Energy	142.92	1396.387	.661	.979
Q3 Energy	142.96	1390.210	.656	.979
Q4 Family	141.94	1385.180	.709	.979
Q5 Family	142.81	1375.595	.690	.979
Q6 Family	143.28	1393.105	.610	.979
Q7 Language	141.74	1396.591	.734	.979
Q8 Language	141.54	1391.688	.707	.979
Q9 Language	141.76	1393.394	.707	.979
Q10 Language	141.86	1390.685	.690	.979
Q11 Language	141.82	1392.347	.707	.979
Q12 Mobility	142.58	1397.683	.752	.979
Q13 Mobility	142.50	1388.394	.785	.978
Q14 Mobility	142.56	1379.405	.813	.978
Q15 Mobility	142.42	1386.500	.753	.978
Q16 Mobility	142.32	1378.925	.807	.978
Q17 Mobility	142.38	1381.646	.790	.978
Q18 Mood	142.36	1376.938	.736	.978
Q19 Mood	142.11	1393.875	.590	.979
Q20 Mood	142.76	1382.465	.699	.979
Q21 Mood	142.53	1376.168	.796	.978
Q22 Mood	141.67	1388.620	.665	.979
Q23 Personality	142.63	1394.350	.515	.979
Q24 Personality	142.21	1388.083	.624	.979
Q25 Personality	142.44	1380.448	.667	.979
Q26 Self Care	143.21	1384.421	.766	.978
Q27 Self Care	142.18	1387.699	.728	.978
Q28 Self Care	142.49	1377.718	.787	.978
Q29 Self Care	143.00	1378.986	.844	.978
Q30 Self Care	142.35	1381.554	.764	.978
Q31 Social Roles	143.07	1395.953	.748	.978
Q32 Social Roles	143.10	1390.033	.788	.978
Q33 Social Roles	143.04	1389.083	.727	.979
Q34 Social Roles	142.44	1392.560	.588	.979
Q35 Social Roles	143.53	1401.379	.638	.979
Q36 Thinking	142.46	1371.519	.759	.978
Q37 Thinking	142.49	1380.620	.670	.974
Q38 Thinking	141.43	1406.192	.525	.979
Q39 UE function	142.61	1398.382	.598	.979
Q40 UE function	142.26	1394.084	.639	.969
Q41 UE function	142.40	1388.666	.670	.979
Q42 UE function	142.08	1388.556	.702	.975
Q43 UE function	142.85	1386.554	.773	.978
Q44 Vision	141.06	1405.546	.571	.979
Q45 Vision	141.18	1405.136	.507	.979
Q46 Vision	141.00	1404.845	.606	.979
Q47 Work Productivity	143.11	1384.213	.742	.958
Q48 Work Productivity	143.00	1396.873	.700	.958
Q49 Work Productivity	143.43	1393.854	.722	.958

### Convergent validity

Two hundred and forty-five respondents completed both SSQoL-Am and SF-36-Am to evaluate the convergent validity of the SSQoL-Am. The Spearman’s Rho correlation showed that the mobility and self-care domains had a strong correlation with the Physical functioning domains of SF-36 with rho = 0.70 and 0.72, p < 0.01, respectively. The correlation between the mental health domain on the Amharic version of SF-36 and the mood and personality domain of the SSQOL-Am demonstrated moderate correlation (*r*ho = 0.49 and 0.58) p < 0.001, respectively. The total SSQOL-Am score showed a statistically significant strong correlation (rho = 0.78) with the overall SF-36 Amharic version in Table 5.

**Table 5:** Spearman’s correlation of SSQoL-Am with the sub-scales of SF-36 (n = 245), 2021

SSQoL domain	Comparison SF-36 subscales	spearman’s rho	r^2^	P
Energy	Vitality	0.35	0.41	< 0.001
Family	Physical, emotional limitation	0.35, 0.28	0.32	< 0.001
Mobility	Physical function	0.70	0.57	< 0.001
Self-care	Physical function	0.72	0.61	< 0.001
Social	Social function	0.43	0.21	< 0.01
Mood	Mental health	0.48	0.39	< 0.001
Personality	Mental health	0.58	0.22	< 0.01
Work	Physical limitation	0.49	0.31	< 0.001

*SF-36* Short-form 36 health survey, *SSQoL* Stroke Specific Quality of Life

### Factor analysis

**Fig. 2:**
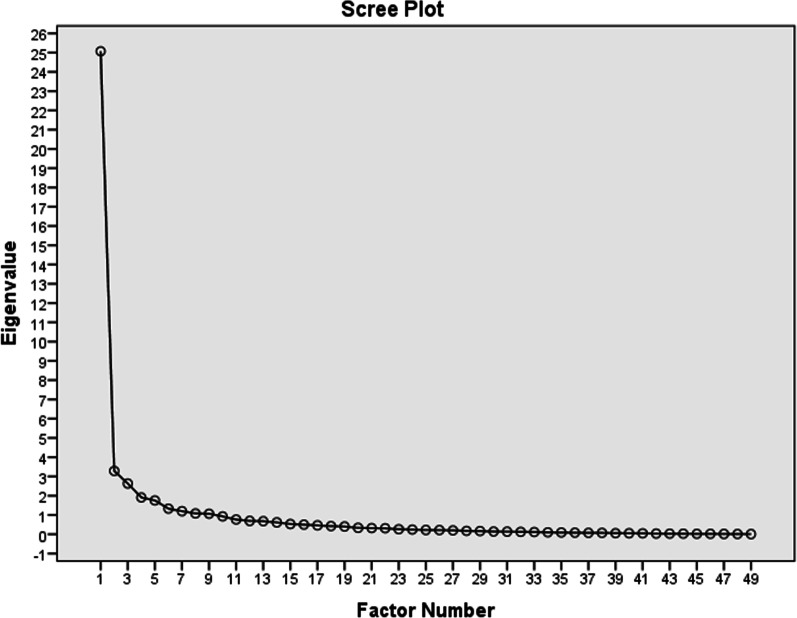
Scree plot indicating factor loading for SSQOL-Am

The CFA of SSQoL of two-factor model assumptions with modification of indices failed to support the priori hypothesis (Additional file 2). To determine the dimensionality of SSQoL-Am a stepwise EFA approach was performed with Kaiser Meyer Olkin (KMO) and Bartlett’s criteria with a retention rule of an Eigenvalue > 1. Besides, a scree plot was visually inspected (Fig. 2). An initial EFA analysis with the Maximum likelihood using the Varimax rotation model, factor loading principle 0.4, resulted in a 9-factor structure with KMO = 0.854. Chi-square = 1973.13, p < 0.001, explaining 80.245 variance with Eigenvalue > 1 Table 6. However, two items (SSQoL-Am # 5, and # 21) in the initial communalities showed factor loading < 0.3. Hence, these two items were removed and the next EFA analysis with the remaining 47 items in a 9-factor structure with an improved KMO = 0.869, Chi-square = 1786.3, p < 0.001. The goodness of fit test indicated a non-significant value (Chi-square = 152.63, df = 117.64, p = 0.124). These values indicate that SSQoL-Am is predominantly a one-factor and secondary nine-factor structure. The first factor explained the variance of 50.9% with an eigenvalue of 23.9. The nine factors explained a total variance of 80.75%.

**Table 6:** Factor loading on the Maximum Likelihood with Varimax rotation for 47 items of SSQoL-Am

	Factors								
	1	2	3	4	5	6	7	8	9
Q# 40 UE function	.836								
Q# 39 UE function	.826								
Q# 42 UE function	.822								
Q# 41 UE function	.821								
Q# 27 Self-Care	.723								
Q# 30 Self-Care	.639								
Q# 43 UE function	.635								
Q# 28 Self-Care	.618								
Q# 29 Self-Care	.522								
Q# 9 Language		.828							
Q# 10 Language		.827							
Q# 11 Language		.809							
Q# 8 Language		.763							
Q# 7 Language		.742							
Q# 49 Work			.800						
Q# 47 Work			.758						
Q4# 8 Work			.729						
Q# 35 Social role			.578						
Q# 6 Family			.556						
Q# 26 Self-Care			.496						
Q# 31 Social role				.653					
Q# 32 Social role				.647					
Q# 13 Mobility				.626					
Q# 33 Social role				.609					
Q# 14 Mobility				.547					
Q# 16 Mobility				.545					
Q# 17 Mobility				.526					
Q# 15 Mobility				.519					
Q# 2 Energy					.737				
Q# 1 Energy					.643				
Q# 3 Energy					.622				
Q# 4 Family					.520				
Q# 12 Mobility					.491				
Q# 45 Vision						.881			
Q# 44 Vision						.875			
Q# 46 Vision						.809			
Q# 19 Mood							.791		
Q# 22 Mood							.595		
Q# 18 Mood							.558		
Q# 34 Social roles							.539		
Q# 20 Mood							.513		
Q#23 Personality								.854	
Q#24 Personality								.775	
Q#25 Personality								.652	
Q#38 Thinking									.700
Q#37 Thinking									.619
Q#36 Thinking									.504
Eigen value of factor	25.1	3.3	2.6	1.9	1.8	1.3	1.2	1.1	1.1
% of variance explained	51.2	6.7	5.3	3.9	3.6	2.7	2.4	2.2	2.2

KMO = 0.869, χ^2^ = 1786.3, only factors loading > 0.4 is indicated. KMO: Kaiser–Meyer–Olkin measure of sampling adequacy; Χ^2^: Barlett’s test of sphericity tested with Chi-square; ***p < 0.001; Extraction method: Maximum Likelihood; Rotation method: Varimax

## Discussion

This study presents the results of the first cross-cultural translation, cultural adaptation, and evaluation of the psychometric properties of the SSQoL among the Amharic-speaking post-stroke survivors in Ethiopia. Amharic speakers account for 29.3% of the country’s population, with an estimate of 34 million native speakers and over 4 million second-language speakers of this Amharic [48].

The translation, cultural adaptation, and synthesis of SSQoL-Am was easy, acceptable by Amharic speaking stroke survivors, and is ready for use in the local settings. The evaluation of the acceptability of the SSQoL-Am showed that all the domains were below the threshold reported for floor effects (20%). However, the ceiling effect of the vision domain was higher than the threshold reported [26]. Previous studies in the United States of America (63%), Denmark (63.8%), Norway (62.7%), and Germany (51.9%) reported higher ceiling effects exceeding the recommended 20% which is considered poor [10, 14, 18, 30]. The variance and higher ceiling effects of vision domain between the studies could be attributed to the difference in time elapsed between the stroke event and administration of the tool, timing of the questionnaire implementation, types, severity, and duration of stroke among the participants' population. Further, had the Amharic speaking stroke survivors taken the retest questionnaire after a substantial period, they might have achieved adequate recovery by that time.

In the Amharic version of SSQoL, 10 of the 12 domains exceeded Cronbach’s alpha of 0.8, revealing that the items within the domains measured the same concept. This is similar to the findings reported by the original version by Williams et al. and other studies. The healthy Cronbach’s alpha of SSQoL-Am explains the adequacy of sample size as discussed by the Muus et al. work [14]. The SSQoL-Am displayed good test–retest stability as observed by the correlation between 0.83 and 0.92 on SSQoL-Am domains, which was taken one week after the first test. The Danish and the American version of SSQoL reported test–retest correlations of 0.99 and 0.92, the re-administration period was 1 week and 2 h, respectively [10, 14]. In contrast, the German version [18] had an assessment interval of 1 year and reported test–retest correlations of 0.69, suggesting that the longer test–retest interval affected the correlation and stability of the tool.

The higher test–retest reliability among our respondents might be related to a possible short recall time of one week. The Bland–Altman plot suggests a good agreement between the measures. The Cronbach’s alpha reported in this study is higher than studies in Saudi Arabia, the United States of America, and Mexico. This could be due to the difference in the sample size. For instance, 245 respondents in this study compared to 147, 34, and 31 stroke survivors in Saudi Arabia [11], the United States of America [10], and Mexico [20], respectively. Thus, the observed difference in Cronbach’s alpha between studies is attributed to items studied, sample size, and heterogeneity of respondents. Further, the present study included stroke survivors from multicenter, with broader chronicity and severity. In contrast, the study in the USA recruited one-month post-stroke subjects. Our results are consistent with the internal consistency of SSQoL reported by the Danish, Brazil, Turkish, and Yoruba version [12, 14, 18, 22].

The SEM and MDC provides an indication of the absolute reliability of a tool and genuine agreement of repeated measurements [49]. The narrow MDC value and small SEM in the present study suggest that SSQoL-Am is a stable measure across repeated administration. Further, the strong reliability, low SEM (< 0.4) scores in most of the domains in the present study indicate that SSQoL-Am is a very reliable tool for clinical and researching HRQOL among stroke survivors. A minor degree of the change in the domain score reflects a true change in the construct, and a change score of 1 SEM might indicate a minimal clinical difference [50]. The domain-wise SEM observed in this study is consistent with the Norwegian version of SSQoL [29].

In this study, the content validity of SSQoL-Am was conducted by a qualitative cognitive debriefing. The convergent validity was established by conducting a correlation test of the tool with the sub scales SF-36 tool [51]. The findings of cognitive debriefing suggest that SSQoL-Am content of interest (Health-related QOL) is captured, participants understood the content, and the concept being measured is easy to understand by Amharic speaking Stroke survivors. As hypothesized, the construct (convergent) validity of SSQoL-Am is reflected in its significant strong to moderate correlation of mobility and self-care domains with the physical functioning sub-scale of SF-36. A similar strong correlation between these domains of SSQL and SF-36 findings have been reported in the original version and other previous studies [10, 11, 29]. This positive correlation may be explained by the relationship between self-care needs and physical functioning abilities in conditions like stroke. In addition, the level of education, family support, and living standards of the participants can be play role in the construct validity of the SSQoL-Am tool.

The construct validity scores (r^2^) on the SSQoL-Am ranged between 0.21 and 0.61 (Table 4), which suggests an adequate linear relationship between most of the SSQoL-Am domains and then compared to SF-36 subscales. This finding is similar to the linear relationship reported by Muss et al. and Williams et al. [10, 14]. But, the social and personality domain of the SSQoL-Am tool had a low linear relationship with the social function and mental health subscales of SF-36, than reported by Williams et al., Odetunde et al., and Muus et al. [10, 14, 17]. This difference can be explained by the variance in elapsed time between the stroke event and administration of the questionnaire, longer the recovery time better the social adaptation. Further, the personality domain attempts to predict human behavior, which varies widely across cultures. As noted by Muus et al., the item ‘I had sex less often than I would like’ (*Social role domain*) had been reported to have the largest missing response. The sensitivity or private nature of items like this could have caused disagreements. The mobility and self-care domains of SSQoL-Am had good agreement with SF-36 (r^2^ 0.57, 0.61 respectively) in this study. These findings are similar to several versions of SSQoL questionnaires with r^2^ ranging between 0.41 and 0.62, suggesting that SSQoL-Am can measure patient mobility and self-care, similar to the SF-36 tool.

Ethiopians are socially more interrelated and social activities are one of priority of their life (reference). The energy domain had a weak but positive correlation with the SF-36 vitality subscale was 0.35, which is higher than what was reported in Mexico (0.08), and Saudi Arabia (0.25); however, the findings are lower than that reported by the United States of America (0.51) and Denmark (0.52) [10, 11, 14, 20]. This difference probably indicates that the established scale is more specifically attributed to translation in questionnaires, cultural differences, and duration of the condition. The SSQOL-Am total score demonstrated a significantly strong correlation with the total score of SF-36 (rho = 0.74, p < 0.001) which was higher than reported in the original tool developer (rho = 0.65, p < 0.001) (14). The overall higher agreement could be attributed to variations in demographics, absence of homeless, people living in organizations in this study, and test–retest duration. Nevertheless, the items of SSQoL-Am domains individually demonstrated a good level of convergent validity (> 0.50) except for the social domain, which had a relatively weak correlation between items and other domains. The weak correlation could be partly explained by the discriminant validity of the tool.

The CFA of SSQoL-Am did not conform to the proposed theory of two-factor structure. The EFA indicated that SSQoL-Am is a predominately a one-factor and secondary nine-factor structure. The factor loads of items located in each factor were found to be moderate and the variability in the number factors explained in this study and the results from other settings [18, 21] could be attributed to the perceptual differences, and subjective nature of the items in SSQoL. This finding suggests the need for further construct evaluations of the SSQoL-Am in Ethiopia. Nevertheless, more importantly all the items of upper extremity functions and 4 out of 5 items of self-care fell into one sub-dimension which almost accords conceptually with the sub-dimension intended by the original version. The significance of our factor analysis is that SSQoL-Am should be considered a tool that may assess stroke related QoL with potentially other underlying constructs when used in research or clinical settings in the Ethiopian stroke survivors.

The generalizability and interpretation of the findings of this study warrant few considerations; only adult stroke survivors were included, the responses to SSQOL-Am might be influenced by severity of symptoms, chronicity of stroke, quality of health-care, and extent of after-effect. Though the study centers are public hospitals and offers acceptable standard rehabilitation services, due to the difference in facilities available between the hospitals, health seeking behavior, utilization of health care, and accessibility the representation of rural stroke survivors seems to low and put forth a probable social bias and variability of the findings. Our study has few limitations. There are higher proportion of ischemic stroke survivors in this study, the different outcomes influenced by the severity of the disease, and about 23% of Amharic speakers in Ethiopia may limit the generalizability of the findings to the entire country though. The chronicity of stroke among participants was significantly different at the baseline. It is understood that the stroke patients at different phases of the post-stroke recovery may report and perceive different HRQoL concerns. It is not clear if the SSQoL-Am demonstrated that validity. Finally, pilot testing and cognitive debriefing were conducted with a small sample. Hence, future studies should address these gaps and the findings of this study should be interpreted with caution based on its limitations.

## Conclusion

Decreased quality of life is often the negative consequence of stroke. The SSQoL is a reliable, valid, and very useful tool that can help evaluate patients QOL. The English version of SSQoL was successfully translated and adapted to the Amharic language. The Amharic version of the SSQoL-Am scale is a tool with good psychometric properties and adequately supported construct validity. It appears to be a suitable tool for use in future epidemiological studies and clinical studies among Amharic speaking Stroke survivors. It is recommended to translate and valid the SSQoL in other languages in Africa to improve response quality of the stroke survivors QoL.

## Supplementary Information

**Additional file 1**: Amharic version of SSQoL-questionnaire.

**Additional file 2**: Minimal Detectable Changes.

## Data Availability

The study contains all of the study data related to these findings. Requests for more information on the dataset and questions about data sharing should be directed to the correspondence author via dachophysio430@gmail.com.

## Abbreviations

AGFI: Adjusted Goodness of Fit Index
CFI: Comparative Fit Index
DALYs: Disability-Adjusted Life Years
EFA: Exploratory Factor Analysis
GBD: Global Burden of Diseases
GFI: Goodness of Fit Index
HRQoL: Health-Related Quality of Life
LOA: Limit of Agreement
MDC: Minimum Detectable Change
RMSEA: Root Mean Square Error of Approximation
SEM: Standard Error of Measurement
SSQOL: Stroke-Specific Quality of Life
