# Supplementary material for: Cross-cultural translation, adaptation, and validation of the stroke-specific quality of life (SSQOL) scale 2.0 into Amharic language
Source: Health Qual Life Outcomes. 2023 Jan 23;21:7. doi: 10.1186/s12955-023-02092-3 (PMC9869570; doi:10.1186/s12955-023-02092-3)
Supplement: Supplementary file 1 — Additional file 1: Amharic version of SSQoL-questionnaire. [file 12955_2023_2092_MOESM1_ESM.docx]

**Additional file 1: Amharic version of SSQoL-questionnaire**

### **የስትሮክ ልዩ የአኗኗር ሁኔታ መለኪያ**

**ነጥብ አሰጣጥ፡ እያንዳንዱ ነጥብ ከዚህ በታች በተዘረዘረው አገላለፅ መሰረት ይመዘናል**

| ሙሉ በሙሉ እርዳታ (1) | ምንም ማከናወን አልችልም (1) | በጣም እስማማለሁ (1) |
| --- | --- | --- |
| ብዙ እርዳታ (2) | በብዙ ችግር (2) | እስማማለሁ (2) |
| መካከለኛ እርዳታ (3) | በመካከለኛ ችግር (3) | ገለልተኛ (3) |
| ትንሽ እርዳታ (4) | በአነስተኛ ችግር (4) | አልስማማም (4) |
| ምንም እርዳታ አያስፈልግም (5) | ያለምንም ችግር (5) | በጣም አልስማማም (5) |

**መመሪያዎች**

በስትሮክ ልጎዱ በሚችሉ አንዳንድ እንቅስቃሴዎች ወይም ስሜቶች እንዴት እንደሚሠሩ ማወቅ እንፈልጋለን፡፡ እያንዳንዱ ጥያቄ ስለ አንድ የተወሰነ እንቅስቃሴ ወይም ስሜት ይጠይቃል።ለእያንዳንዱ ጥያቄ ባለፈው ሳምንት ውስጥ ያ እንቅስቃሴ ወይም ያ ስሜት ለእርስዎ እንዴት እንደነበረ ያስቡ።

ጥያቄዎች በተወሰኑ እንቅስቃሴዎች ላይ ምን ያህል ችግር እንዳለብዎ እንድሁም ምን ያህል መስማማት ወይም አለመስማማት እንዳለቦዎት ይጠየቃሉሁ፡፡ እያንዳንዱ ጥያቄ አንዳንድ ሰዎች ከስትሮከ በኋላ ካጋጠሟቸው ችግሮች ጋር ይያያዛል ፡፡

ባለፈው ሳምንት እንቅስቃሴ ምን ያህል ችግር እንደገጠመዎት በተሻለ ሁኔታ የሚገልጽ አንድ ሳጥን ውስጥ ይምረጡ

| **አቅም** | | |  |  |  |  |  |
| --- | --- | --- | --- | --- | --- | --- | --- |
| ተ.ቁ | |  | በጣም እስማማለሁ | እስማማለሁ | ገለልተኛ | አልስማማም | በጣም አልስማማም |
| 1 | | ብዙዉን ጊዜ የድካም ስሜት ይሰማኝ ነበር | 1 | 2 | 3 | 4 | 5 |
| 2 | | በቀን ውስጥ ስራየን አቁሜ ማረፍ ነበረብኝ | 1 | 2 | 3 | 4 | 5 |
| 3 | | ማከናወን የምፈልገውን ለማከናወን በጣም ደክሞኝ ነበር | 1 | 2 | 3 | 4 | 5 |
| **የቤተሰብ ሚና** | | |  |  |  |  |  |
| ተ.ቁ | | ባለፈው ሳምንት | በጣም እስማማለሁ | እስማማለሁ | ገለልተኛ | አልስማማም | በጣም አልስማማም |
| 1 | | ከቤተሰብ ጋር ተቀላቅዬ መጫወት አልቻኩም | 1 | 2 | 3 | 4 | 5 |
| 2 | | ለቤተሰቤ ሸክም እንደሆንኩ ይሰማኝ ነበር | 1 | 2 | 3 | 4 | 5 |
| 3 | | አካላዊ ሁኔታዬ የግል ሕይወቴ ላይ ተፅዕኖ አሳድሮብኛል | 1 | 2 | 3 | 4 | 5 |
| **ቋንቋ** | | |  |  |  |  |  |
| ተ.ቁ | | ባለፈው ሳምንት | ምንም ማከናወን አልችልም | በብዙ ችግር | በመካከለኛ ችግር | በአነስተኛ ችግር | ያለምንም ችግር |
| 1 | | የመናገር ችግር አጋጥመዎታል? ለምሳሌ የቃላት መቆራረጥ ፣የመንተባተብ ፣የመንቀጥቀጥና ቃላቶችን የመጎተት? | 1 | 2 | 3 | 4 | 5 |
| 2 | | በስልክ በደንብ ጥርት አድርጎ የመናገር ችግር ገጥመዎታል? | 1 | 2 | 3 | 4 | 5 |
| 3 | | ሌሎች ሰዎች የእርስዎን ንግግር ለመረዳት ተቸግረዋል? | 1 | 2 | 3 | 4 | 5 |
| 4 | | መናገር የፈለጉትን ለመናገር ቃላት በመፈለግ ተቸግረዋል? | 1 | 2 | 3 | 4 | 5 |
| 5 | | ሌሎች እንዲረድዎት ንግግርዎን መድገም ይጠበቅቦት ነበር? | 1 | 2 | 3 | 4 | 5 |
| **እንቅስቃሴ** | | |  |  |  |  |  |
| ተ.ቁ | | ባለፈው ሳምንት | ምንም መከናወን አልችልም | በብዙ ችግር | በመካከለኛ ችግር | በአነስተኛ ችግር | ያለምንም ችግር |
| 1 | | የመራመድ ችግር ገጥሞታል? (ታካሚው ለመራመድ የማይችሉ ከሆነ ወደ ጥያቄ ቁጥር 4 ይሄዱና ጥያቄ ቁጥር 2-3 እንደ 1 ደረጃ ይሰጡ) | 1 | 2 | 3 | 4 | 5 |
| 2 | | ወደ የሆነ ነገር ለመድረስ ሲጎነበሱ ሚዛንዎትን አጥተዋል? | 1 | 2 | 3 | 4 | 5 |
| 3 | | ደረጃ ለመውጣት ተቸግረዋል? | 1 | 2 | 3 | 4 | 5 |
| 4 | | በሚራመዱበት ወይንም ተሽከርካሪ ወንበር በሚጠቀሙበት ጊዜ ከሚፈልጉት በላይ መቆም እና ማረፍ ነበረቦት? | 1 | 2 | 3 | 4 | 5 |
| 5 | | በሚቆሙበት ጊዜ ተቸግረዋል? | 1 | 2 | 3 | 4 | 5 |
| 6 | | ከወንበር ለመነሳት ተቸግረዋል? | 1 | 2 | 3 | 4 | 5 |
| **ስሜት** | | | | | | | |
| ተ.ቁ | | ያለፈው ሳምንት | በጣም እስማማለሁ | እስማማለሁ | ገለልተኛ | አልስማማም | በጣም አልስማማም |
| 1 | | ስለ ወደፊት ተስፋ ቆረጨ ነበር | 1 | 2 | 3 | 4 | 5 |
| 2 | | ለሌሎች ሰዎች ወይም እንቅስቃሴዎች ፍላጎት አልነበረኝም | 1 | 2 | 3 | 4 | 5 |
| 3 | | ከሌሎች ሰዎች እንደሸሸሁ ይሰማኝ ነበር | 1 | 2 | 3 | 4 | 5 |
| 4 | | በራሴ ላይ ያለኝ መተማመን አነስተኛ ነበር | 1 | 2 | 3 | 4 | 5 |
| 5 | | የምግብ ፍላጎት አልነበረኝም | 1 | 2 | 3 | 4 | 5 |
| **ስብዕና** | | | | | | | |
| ተ.ቁ | | ያለፈው ሳምንት | በጣም እስማማለሁ | እስማማለሁ | ገለልተኛ | አልስማማም | በጣም አልስማማም |
| 1 | | ብስጩ ነበረኩ | 1 | 2 | 3 | 4 | 5 |
| 2 | | በሌሎች ሰዎች ላይ ትግስት አልነበረኝም | 1 | 2 | 3 | 4 | 5 |
| 3 | | ባህሪዬ ተቀይሯል | 1 | 2 | 3 | 4 | 5 |
| **ራስን መጠበቅ** | | | | | | | |
| ተ.ቁ | | ያለፈው ሳምንት | ሙሉ በሙሉ እርዳታ | ብዙ እርዳታ | መካከለኛ እርዳታ | ትንሽ እርዳታ | ምንም እርዳታ አያስፈልግም |
| 1 | | ምግብ ለማዘጋጀት እርዳታ ይፈልጉ ነበር? | 1 | 2 | 3 | 4 | 5 |
| 2 | | ለመመገብ እርዳታ ይፈልጉ ነበር? ለምሳሌ ምግቡን ለመቁረጥና እና ለማዘጋጀት? | 1 | 2 | 3 | 4 | 5 |
| 3 | | ለመልበስ እርዳታ ይፈልጉ ነበር? ለምሳሌ ጫማ ወይም ካልሲ ለማጥለቅ ቁልፍ ለመቆለፍ ወይም ዚፕ ለመሸርገግ? | 1 | 2 | 3 | 4 | 5 |
| 4 | | ገላዎን ለመታጠብ ወይንም ሻወር ለመውሰድ እርዳታ ይፈልጉ ነበር? | 1 | 2 | 3 | 4 | 5 |
| 5 | | ሸንት ቤት ለመጠቀም እርዳታ ይፈልጉ ነበር? | 1 | 2 | 3 | 4 | 5 |
| ማህበራዊ ሚናዎች | | | | | | | |
| ተ.ቁ | | ያለፈው ሳምንት | በጣም እስማማለሁ | እስማማለሁ | ገለልተኛ | አልስማማም | በጣም አልስማማም |
| 1 | | ብዙ ጊዜ የፈለኩትን ያክል መውጣት አልችልም ነበር | 1 | 2 | 3 | 4 | 5 |
| 2 | | የሚያስደስቱኝን እና የሚያዝናኑኝን ነገሮች የምፈልገዉ ጊዜ ያክል ማደረግ አልቻልኩም ነበር | 1 | 2 | 3 | 4 | 5 |
| 3 | | ብዙ ጓደኞቼን የፈለኩትን ያክል ማግኘት አልቻልኩም ነበር | 1 | 2 | 3 | 4 | 5 |
| 4 | | ከምፈልገው ጊዜ ባነሰ ግብረስጋ ግንኙነት እፈፅም ነበር | 1 | 2 | 3 | 4 | 5 |
| 5 | | አካላዊ ሁኔታዬ በማህበራዊ ሕይወቴ ላይ ተፅዕኖ አሳድሮብኛል | 1 | 2 | 3 | 4 | 5 |
| **አስተሳሰብ** | | | | | | | |
| ተ.ቁ | | ያለፈው ሳምንት | በጣም እስማማለሁ | እስማማለሁ | ገለልተኛ | አልስማማም | በጣም አልስማማም |
| 1 | | ትኩረት ለመስጠት እቸገር ነበር | 1 | 2 | 3 | 4 | 5 |
| 2 | | ነገሮችን ለማስታወስ ተቸግሬለሁ | 1 | 2 | 3 | 4 | 5 |
| 3 | | ነገሮችን ለማስታወስ መፃፍ ነበረብኝ | 1 | 2 | 3 | 4 | 5 |
| **የላይኛው ክንፈ አካል ተግባር** | | | | | | | |
| ተ.ቁ | | ያለፈው ሳምንት | ምንም ማከናወን አልችልም | በብዙ ችግር | በመካከለኛ ችግር | በአነስተኛ ችግር | ያለምንም ችግር |
| 1 | | ለመፃፍ ወይም ለመተየብ ተቸግረዋል? | 1 | 2 | 3 | 4 | 5 |
| 2 | | ካልሲ ለማጥለቅ ተቸግረዋል? | 1 | 2 | 3 | 4 | 5 |
| 3 | | የልብስ ቁልፍ ለመቆለፍ ተቸግረዋል? | 1 | 2 | 3 | 4 | 5 |
| 4 | | ዚፐር ለመሸርገግ ተቸግረዋል? | 1 | 2 | 3 | 4 | 5 |
| 5 | | ጀርካን ለመክፈት ተቸግረዋል? | 1 | 2 | 3 | 4 | 5 |
| **የማየት ችሎታ** | | |  |  |  |  |  |
| ተቁ | | ያለፈው ሳምንት | ምንም ማከናወን አልችልም | በብዙ ችግር | በመካከለኛ ችግር | በአነስተኛ ችግር | ያለምንም ችግር |
| 1 | | ~~በ~~ቴሌቭዥን በደንብ ለማየት እና ለመዝናናት የዕይታ ችግር ገጥመዎታል? | 1 | 2 | 3 | 4 | 5 |
| 2 | | በዕይታ ችግር ምክንያት የሚፈልጉትን ነገሮች ላይ ለመድረስ ተቸግረዋል? | 1 | 2 | 3 | 4 | 5 |
| 3 | | በአንድ ጎን ያለን ነገር ለማየት ተቸግረዋል? | 1 | 2 | 3 | 4 | 5 |
| **ስራ /ምርታማነት** | | |  |  |  |  |  |
| ተ.ቁ | ያለፈው ሳምንት | | ምንም ማከናወን አልችልም | በብዙ ችግር | በመካከለኛ ችግር | በአነስተኛ ችግር | ያለምንም ችግር |
| 1 | በቤት ዉስጥ የዕለት ተዕለት ስራዎች ለመስራት ተቸግረዋል? | | 1 | 2 | 3 | 4 | 5 |
| 2 | የጀመሩትን ስራ ለመጨረስ ተቸግረዋል? | | 1 | 2 | 3 | 4 | 5 |
| 3 | በፊት ሲሰሩ የነበረውን ሰራ ለማከወን ተቸግረዋል? | | 1 | 2 | 3 | 4 | 5 |
| **ውጤት** | | |  |  |  |  |  |
| **ጠቅላላ ውጤት** | | |  |  |  |  |  |
