# Supplementary material for: Cross-cultural translation, adaptation, and validation of the stroke-specific quality of life (SSQOL) scale 2.0 into Amharic language
Source: Health Qual Life Outcomes. 2023 Jan 23;21:7. doi: 10.1186/s12955-023-02092-3 (PMC9869570; doi:10.1186/s12955-023-02092-3)
Supplement: Supplementary file 2 — Additional file 2: Minimal Detectable Changes. [file 12955_2023_2092_MOESM2_ESM.docx]

**Additional tables**

**Table I**

A priori hypothesis to assess convergent construct validity between SSQoL-Am and SF-36-Am subscales in Amharic speaking stroke survivors based on COSMIN guideline (n = 245)

| **Outcomes** | **Hypothesized**  **Correlations^*^** | **Estimated**  **Correlations*** | **Hypothesis acceptance** |
| --- | --- | --- | --- |
| **SF-36-Am subscales** |  |  |  |
| Vitality | 0.00-0.69 | 0.35 | Yes |
| Physical, emotional limitation | 0.00-0.69 | 0.35,0.28 | Yes |
| Physical function | 0.00-0.69 | 0.70 | Yes |
| Physical function | 0.00-0.69 | 0.72 | Yes |
| Social function | 0.00-0.69 | 0.43 | Yes |
| Mental health | 0.00-0.69 | 0.48 | Yes |
| Mental health | 0.00-0.69 | 0.58 | Yes |
| SF-36, physical limitation | 0.00-0.69 | 0.49 | Yes |
| Number of met hypothesis (%) | 8/8 (100) | 0.74, 0.78 |  |

*Positive correlations, SF-36-Am Amharic version of the short form health survey, SSQoL-Am Amharic version of the Stroke Specific Quality of Life scale.

**Table II:** Standard deviation, Test-retest reliability (ICC), Standard Error of Measurement (SEM), and 95% Minimum Detectable Change (MDC) of SSQoL-Am (N=72)

| **S.no** | **Item** | **meanT1** | **meanT2** | **SE** | **SD** | **ICC** | **SEM** | **MCD_95_** |
| --- | --- | --- | --- | --- | --- | --- | --- | --- |
| 1 | Q1 Energy | 2.43 | 2.57 | 0.13 | 1.07 | 0.871 | 0.38 | 1.07 |
| 2 | Q2 Energy | 2.46 | 2.47 | 0.12 | 1.03 | 0.852 | 0.39 | 1.10 |
| 3 | Q3 Energy | 2.38 | 2.43 | 0.13 | 1.11 | 0.843 | 0.44 | 1.22 |
| 4 | Q4 Family | 3.25 | 3.44 | 0.14 | 1.17 | 0.848 | 0.46 | 1.27 |
| 5 | Q5 Family | 2.92 | 2.58 | 0.15 | 1.30 | 0.871 | 0.46 | 1.30 |
| 6 | Q6 Family | 2.51 | 2.11 | 0.14 | 1.20 | 0.63 | 0.47 | 2.03 |
| 7 | Q7 Language | 3.47 | 3.65 | 0.12 | 1.02 | 0.82 | 0.43 | 1.20 |
| 8 | Q8 Language | 3.42 | 3.85 | 0.13 | 1.12 | 0.823 | 0.47 | 1.31 |
| 9 | Q9 Language | 3.47 | 3.63 | 0.13 | 1.11 | 0.837 | 0.45 | 1.25 |
| 10 | Q10 Language | 3.42 | 3.53 | 0.13 | 1.12 | 0.864 | 0.41 | 1.15 |
| 11 | Q11 Language | 3.43 | 3.57 | 0.13 | 1.10 | 0.845 | 0.43 | 1.21 |
| 12 | Q12 Mobility | 2.89 | 2.81 | 0.12 | 0.98 | 0.831 | 0.40 | 1.12 |
| 13 | Q13 Mobility | 2.94 | 2.89 | 0.12 | 1.00 | 0.87 | 0.36 | 1.00 |
| 14 | Q14 Mobility | 2.86 | 2.83 | 0.14 | 1.14 | 0.901 | 0.36 | 1.00 |
| 15 | Q15 Mobility | 3.04 | 2.97 | 0.13 | 1.12 | 0.834 | 0.45 | 1.26 |
| 16 | Q16 Mobility | 3.04 | 3.07 | 0.13 | 1.14 | 0.858 | 0.43 | 1.19 |
| 17 | Q17 Mobility | 2.94 | 3.01 | 0.13 | 1.12 | 0.836 | 0.45 | 1.26 |
| 18 | Q18 Mood | 2.75 | 3.03 | 0.16 | 1.28 | 0.928 | 0.34 | 0.95 |
| 19 | Q19 Mood | 2.82 | 3.28 | 0.14 | 1.19 | 0.795 | 0.51 | 1.50 |
| 20 | Q20 Mood | 2.9 | 2.63 | 0.14 | 1.18 | 0.874 | 0.41 | 1.16 |
| 21 | Q21 Mood | 2.71 | 2.86 | 0.14 | 1.17 | 0.847 | 0.45 | 1.27 |
| 22 | Q22 Mood | 3.11 | 3.72 | 0.14 | 1.22 | 0.685 | 0.48 | 1.90 |
| 23 | Q23 Personality | 2.65 | 2.76 | 0.15 | 1.26 | 0.904 | 0.39 | 1.08 |
| 24 | Q24 Personality | 2.74 | 3.18 | 0.14 | 1.21 | 0.816 | 0.42 | 1.44 |
| 25 | Q25 Personality | 2.76 | 2.94 | 0.15 | 1.25 | 0.901 | 0.39 | 1.09 |
| 26 | Q26 Self Care | 2.22 | 2.18 | 0.13 | 1.07 | 0.867 | 0.39 | 1.08 |
| 27 | Q27 Self Care | 2.89 | 3.21 | 0.14 | 1.07 | 0.839 | 0.43 | 1.19 |
| 28 | Q28 Self Care | 2.9 | 2.9 | 0.14 | 1.22 | 0.91 | 0.36 | 1.02 |
| 29 | Q29 Self Care | 2.6 | 2.39 | 0.13 | 1.14 | 0.858 | 0.43 | 1.19 |
| 30 | Q30 Self Care | 2.83 | 3.04 | 0.14 | 1.18 | 0.884 | 0.40 | 1.12 |
| 31 | Q31 Social Roles | 2.26 | 2.32 | 0.11 | 0.94 | 0.898 | 0.30 | 0.84 |
| 32 | Q32 Social Roles | 2.36 | 2.29 | 0.12 | 0.98 | 0.91 | 0.29 | 0.82 |
| 33 | Q33 Social Roles | 2.29 | 2.35 | 0.13 | 1.07 | 0.942 | 0.25 | 0.71 |
| 34 | Q34 Social Roles | 2.93 | 2.94 | 0.14 | 1.22 | 0.795 | 0.50 | 1.54 |
| 35 | Q35 Social Roles | 2.21 | 1.86 | 0.12 | 1.03 | 0.673 | 0.49 | 1.65 |
| 36 | Q36 Thinking | 2.83 | 2.93 | 0.15 | 1.26 | 0.938 | 0.31 | 0.87 |
| 37 | Q37 Thinking | 2.86 | 2.9 | 0.15 | 1.26 | 0.903 | 0.39 | 1.10 |
| 38 | Q38 Thinking | 3.32 | 3.96 | 0.13 | 1.11 | 0.626 | 0.38 | 1.89 |
| 39 | Q39 UE function | 2.78 | 2.78 | 0.13 | 1.09 | 0.862 | 0.40 | 1.13 |
| 40 | Q40 UE function | 2.86 | 3.13 | 0.14 | 1.15 | 0.872 | 0.41 | 1.15 |
| 41 | Q41 UE function | 2.93 | 2.99 | 0.14 | 1.14 | 0.832 | 0.46 | 1.30 |
| 42 | Q42 UE function | 3.08 | 3.31 | 0.14 | 1.18 | 0.835 | 0.47 | 1.33 |
| 43 | Q43 UE function | 2.76 | 2.54 | 0.13 | 1.10 | 0.831 | 0.45 | 1.26 |
| 44 | Q44 Vision | 4.36 | 4.33 | 0.11 | 0.96 | 0.821 | 0.40 | 1.13 |
| 45 | Q45 Vision | 4.15 | 4.21 | 0.13 | 1.12 | 0.842 | 0.44 | 1.24 |
| 46 | Q46 Vision | 4.36 | 4.39 | 0.11 | 0.94 | 0.806 | 0.41 | 1.15 |
| 47 | Q47 Work Productivity | 2.38 | 2.28 | 0.13 | 1.09 | 0.872 | 0.39 | 1.09 |
| 48 | Q48 Work Productivity | 2.35 | 2.39 | 0.12 | 1.01 | 0.89 | 0.33 | 0.93 |
| 49 | Q49 Work Productivity | 2.15 | 1.96 | 0.12 | 1.06 | 0.821 | 0.44 | 1.24 |
